# Combined Effect of Low-Temperature Stress and Slightly Acidic Electrolyzed Water (SAEW) on the Microbial Control of Oat Sprout Production

**DOI:** 10.3390/foods14071083

**Published:** 2025-03-21

**Authors:** Shaokang Liu, Hongrui Ren, Lin Chen, Tongjiao Wu, Jianxiong Hao

**Affiliations:** College of Food Science and Biology, Hebei University of Science and Technology, Shijiazhuang 050018, China; 18833106110@163.com (S.L.); rrhr0220@163.com (H.R.); c2537419491@163.com (L.C.)

**Keywords:** oat sprouts, slightly acidic electrolyzed water, disinfectant, microbial control

## Abstract

Sprouts have many advantages, such as high nutritional value and simple cultivation conditions. In recent years, the loss rate of sprouts due to microbial contamination has been as high as 40%, and it is necessary to strengthen microbial control technology to reduce such losses. Current microbial control methods have issues such as a narrow bactericidal spectrum and high cost and contamination, whereas slightly acidic electrolyzed water (SAEW), as a new type of disinfectant, can solve these problems. In the present study, the efficacy of SAEW treatments in the disinfection of oat sprouts during germination and washing was evaluated at 25 °C and 4 °C compared to a TW control group. The results showed that compared to the TW control group, the *Escherichia coli* detection rates were significantly decreased by 2.32, 4.44 and 5.55 log_10_ CFU/g after soaking, undergoing germination for 60 h and washing with SAEW at 4 °C. This indicated that the 4 °C + SAEW treatment had a favorable bactericidal effect on the whole process of oat sprout washing. This result was demonstrated by scanning electron microscopy (SEM). In addition, for natural colony counts in oat sprouts, the 4 °C + SAEW treatment also showed strong bactericidal ability. Therefore, the application of SAEW combined with low temperature stress treatment in sprout production and processing has high potential.

## 1. Introduction

Food safety is an important cornerstone of the global public health system, and its prevention and control risks are present in all aspects of the food production chain [1]. In the field of plant food, sprouts are increasingly favored by people for their unique nutritional value and health benefits [2]; however, they are a high-risk carrier of microbial contamination due to their humid breeding environment and short-cycle growth characteristics. The nutrition-rich matrix formed by these products during seed germination, combined with the suitable temperature of 20–30 °C, provides an ideal environment for the proliferation of foodborne pathogens such as *Salmonella*, *Escherichia coli* (*E. coli*) O157:H7 and *Listeria* [3,4]. In fact, in recent decades, sprout contamination has become an international food safety problem [4,5,6,7]. Outbreaks of disease caused by the consumption of contaminated sprouts have occurred all over the world. For example, in 2005, Canada reported an outbreak of 552 patients linked to *Salmonella* contamination in mung bean sprouts [8]. During 2011, a point source outbreak of infection caused by rare enterohemorrhagic *E. coli* O104:H4-contaminated germinated seeds occurred in Europe [9]. It is worth noting that the contamination risk of different varieties of sprouts present significant differences. Oat sprouts face more complex microbial challenges due to their unique physical and chemical properties. The endosperm of oat sprouts has a high content of β-glucan and soluble proteins, which promotes plant growth [10], while providing a high-quality carbon and nitrogen source for microbial metabolism. More critically, the synergistic effect of the relatively loose cell structure of oat sprout tissue (cell gap up to 15–20 μm) and a neutral pH (6.2–6.8) significantly enhance the biofilm formation capacity of pathogenic bacteria. This special microecological environment, combined with the common raw food habits in its consumption (such as the direct consumption of salads and sandwiches), causes the microbial contamination problem of oat sprouts to comprise both hidden biological hazards and a high risk of exposure, and it is urgent to establish a targeted microbial pollution prevention and control system.

There are many ways to sterilize bean sprouts, such as low-temperature plasma, chemical disinfectants, irradiation techniques and SAEW. Low-temperature plasma technology can achieve efficient sterilization while ensuring food quality [11], but there are limitations, such as a high dependence on equipment and the uniformity of treatment. The use of chemical disinfectants (such as calcium hypochlorite) is relatively inexpensive, but disinfectants are highly corrosive [12] and can cause bacteria to enter a “viable but not culturable” (VBNC) state [13]. Irradiation disinfection is effective in killing a variety of microorganisms, including *Bacillus*, but is expensive and may adversely affect the sensory quality and nutritional content of foods [14]. Slightly acidic electrolyzed water (SAEW) has been widely used in sterilization [15,16]. Although SAEW quickly fades after its production, and in order to use it industrially, it is necessary to produce it directly “on the job” with special equipment, compared with low-temperature plasma technology, SAEW has the advantages of strong penetration, low costs and wide applicability. In comparison with chemical disinfectants, it has the advantages of high efficiency, no pollution and no corrosion [17]. In contrast to irradiation technology, it has the advantages of high safety and simple operation, which makes it one of the most promising disinfectants in the food industry. Previous studies have shown that SAEW can effectively remove pathogenic bacteria such as *E. coli* from the surface of agricultural products without adversely affecting product quality and safety [18]. Moreover, a study on fresh cucumbers showed that SAEW was effective in killing natural colonies and artificially contaminated *E. coli* on the surface of cucumbers [19]. Therefore, applying SAEW for microbial control during the whole process of oat sprout production and washing can help to control the microbial community of oat sprouts, improve their growth quality and stress resistance and lay a solid foundation for high-yield and high-quality oat sprout production. However, the application of SAEW in oat sprouts has not been paid enough attention. There is a lack of in-depth research on both its specific mechanism of action on oat sprouts’ microbial community, as well as the appropriate application concentration, action time and temperature parameters.

In the present study, the effects of low-temperature stress combined with SAEW treatment on microbial control in oat sprout production were investigated. In order to comprehensively evaluate the reduction effect of SAEW on oat sprout microbial communities under two different environmental conditions, an ambient temperature and a low temperature, 25 °C and 4 °C, were selected for the comparative experiments of SAEW treatment, where the reduction in the inoculated bacteria and natural colonies during the whole process of production and the washing of oat sprouts was studied. In this case, to further demonstrate the bactericidal effect of SAEW, sprayed tap water (TW) treatment during germination was used as a control.

## 2. Materials and Methods

### 2.1. Procurement of Oat Seeds

The oat (*Avena sativa*) variety used in this study was Qingyin No. 3, which was produced in Xining City, Qinghai Province, China, in 2024, and all samples were stored at 4 °C for no more than three months from picking to use.

### 2.2. Seed Preparation

All samples were rinsed with running water once before use to remove impurities. Full grain oat seeds with no defects were selected for experimental use.

### 2.3. Preparation of Treatment Solutions

A diaphragm-free electrolysis cell (AQUACIDO NDX-250KMS, OSG Company Ltd., Osaka, Japan) was used to prepare SAEW. Briefly, 4 L of tap water was placed in the cell, and then 10.0 g of CaCl_2_ and 0.5 mL of concentrated HCl were added and thoroughly mixed. After connecting the power supply, the SAEW was produced in the electrolysis cell under a stable current of 1.5 A for 11 min. The pH of the treatment solution was measured with a pH meter (Orion Inc., Reston, VA, USA) and adjusted to 5.90 with a dilute HCl solution. The available chlorine concentration (ACC) in the treated solution was determined using a digital titrator (16900, Hach Company, Loveland, CO, USA). The ACC of the tap water (TW) in the control group was 7.88 and the pH was 7.50.

### 2.4. Preparation of Bacterial Suspension

*Escherichia coli (E. coli)* O157: H7 (ATCC 25922) used in this study was purchased from Beijing Solaibao Biotechnology Co., Ltd. In a sterile environment, the strain was streaked on LB nutrient agar medium and cultured in a biochemical incubator (LRH type, Shanghai Yiheng Scientific Instrument Co., Ltd., Shanghai, China) at 37 °C for 24 h. Single colonies were selected and inoculated into 200 mL of LB broth medium, which was then incubated at 37 °C (160 r/min) in a constant-temperature shaking incubator (ZWY-100H type, Shanghai Zhicheng Analytical Instrument Manufacturing Co., Ltd., Shanghai, China) for 24 h. The bacterial suspension was centrifuged (model GL-20G-Ⅱ, Shanghai Anting Equipment Co., Ltd., Shanghai, China) at 6000 r/min for 10 min, after which the supernatant was discarded. The resulting precipitates were washed twice with 0.85% sterile normal saline and then resuspended in sterile normal saline. The final cell concentration was adjusted to approximately 10⁸ CFU/mL. The bacterial concentration was estimated by measuring the optical density of the suspension at 600 nm using a spectrophotometer (Model UV-500, Shanghai Yuan Analysis Instrument Co., Ltd., Shanghai, China).

### 2.5. Sample Inoculation and Treatments

Oat (sprouts) samples weighing 60 g were submerged into 240 mL of an *E. coli* O157:H7 suspension for 30 min. The inoculated samples were then spread flat in disposable sterile culture dishes and placed in a fume hood (SW-TFG-18, Shanghai Hujing Medical Equipment Co., Ltd., Shanghai, China) maintained at 40–50% humidity. The second-level ventilation device was activated to dry the samples for 15 min, enabling inoculant adhesion to the oat surfaces.

At 25 °C (or 4 °C), the *E. coli* O157:H7-inoculated oat seeds were soaked in SAEW at a 1:5 solid–liquid ratio for 4 h. The soaked seeds were then spread evenly and transferred into a constant temperature/humidity incubator (BIC-400 model, Shanghai Boxun Industrial Co., Ltd., Shanghai, China) for germination under controlled conditions (25 °C or 4 °C, 90% relative humidity) for 60 h. During germination, 1000 mL of treatment solution was sprayed onto the samples at 12 h intervals. Following germination, 10 g samples were collected from each treatment group, soaked in 60 mL of the corresponding solution for 5 min and drained using filter paper. Sampling occurred at three stages: immediately after seed soaking was completed, at 12 h intervals during germination (12, 24, 36, 48 and 60 h) and post-sprout washing.

Samples (10 g) of harvested oat sprouts were then ground in 90 mL of 0.85% sterile physiological saline, sonicated for 1 min cycles (1 min pauses between cycles, repeated 5 times) and stirred at a low speed for 2 min to prepare 10-fold dilutions (10^−1^). Serial dilutions from 10^−2^ to 10^−8^ were prepared by adding 1 mL aliquots to 9 mL of sterile saline. *E. coli* populations were quantified using the dilution coating plate method. Control samples of uninoculated oat seeds underwent identical SAEW treatment protocols at 25 °C (or 4 °C), with natural microbial colonies enumerated using the same dilution spread plate methodology.

### 2.6. Microbiological Analysis

The microbiological analysis was carried out according to the methodology reported by Lei [20], with some modifications.

For post-inoculation *E. coli* counts, the populations of *E. coli* O157:H7 on the oat (sprouts) were determined by selecting five dilutions from 10^−6^ to 10^−8^, each100 μL, which were coated on modified sorbitol MacConkey agar (CT-SMAC, Guangdong Zhongshan Bai Microbial Technology Co., Ltd., Foshan, China) and incubated at 37 °C for 48 h before colony counting.

For the total bacterial count, five dilutions from 10^−2^ to 10^−6^ were selected, each 100 μL, plated on Plate Count Agar (Beijing Aoboxing Biotechnology Co., Ltd., Beijing, China), incubated at 37 °C for 48 h and the colonies were counted. For mold and yeast counts, five dilutions from 10^−2^ to 10^−6^ were selected, each 100 μL, spread on potato dextrose agar plates (Beijing Aoboxing Biotechnology Co., Ltd., Beijing, China), incubated at 28 °C for 48 h and the colonies were counted.

Each dilution was prepared in triplicate. After colony counting, data from the effective dilutions (30–300 CFU/plate) were used to calculate the original microbial concentration using the formula:

(1) $$N=lg(N0\times 10^{x})$$

where N is the bacterial content (lg CFU/g); N0 is the Petri dish count; and x is the dilution factor.

### 2.7. Observation of Scanning Electron Microscopy (SEM)

The oat (sprouts) samples were fixed overnight with 2.5% glutaraldehyde in phosphate-buffered saline. After the dehydration steps with 50%, 70%, 80% and 90% ethanol for 10 min, the samples were dehydrated twice with anhydrous ethanol for 15 min, dehydrated twice with isoamyl acetate for 15 min, coated with goldepalladium and observed under a scanning electron microscope (FEI-X130, Philips Electro Optics Ltd., Eindhoven, The Netherlands). Three evenly distributed fields of view were randomly selected for each sample, and each treatment was repeated in triplicate.

### 2.8. Data Processing and Analysis

Each treatment was performed in triplicate, and the resulting data were calculated as the mean and the standard deviation. The results were analyzed by Duncan’s multiple range test using SPSS software (Statistical Package for the Social Sciences; SPSS Statistics 27, SPSS Inc., Chicago, IL, USA). The statistical significance was set at *p* < 0.05. The graphs were drawn using Origin 2024 drawing software.

## 3. Results and Discussion

### 3.1. Combined Effect of Low Temperature Stress and SAEW on E. coli Artificially Inoculated During Oat Sprout Production

**Figure 1:**
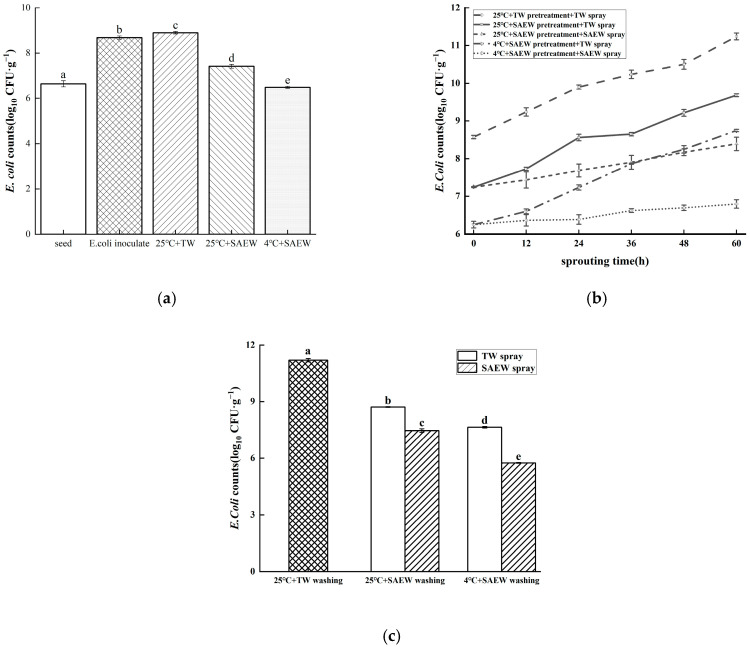
The surviving microbial population of oats inoculated by *E. coli* during the soaking (**a**), germination (**b**) and washing (**c**) stages after being treated with SAEW at different temperatures. The pH and available chlorine concentration (ACC) of SAEW used in the experiment were 5.9 ± 0.1 and 30 mg/L, respectively; the tap water (TW) as the control was local drinking water. The samples should be taken promptly after the completion of seed soaking, during germination (after 12, 24, 36, 48 and 60 h) and after the end of sprout washing. Each value is expressed as the mean ± standard deviation of the three replicates. Different superscripts (a–e) show significant differences in the duration of germination (*p* < 0.05).

The initial microbial populations on the oat seeds, after inoculation and drying in a fume hood, were 8.54 log_10_ CFU/g. As shown in Figure 1a, after soaking at 25 °C + TW, 25 °C + SAEW and 4 °C + SAEW, the *E. coli* O157:H7 populations changed to 8.57 log_10_ CFU/g, 7.24 log_10_ CFU/g and 6.25 log_10_ CFU/g, respectively. The results indicated no significant difference between the oat seeds treated at 25 °C + TW and the initial microbial populations (*p* > 0.05). Although studies have shown that the washing effect of TW can lead to a reduction in the microbial populations on the surface of fresh-cut fruits and vegetables [21], during prolonged immersion, the humid environment provided by tap water can harbor bacteria, as tap water itself carries bacteria [20]. In contrast to the TW control group, SAEW significantly reduced the microbial population (*p* < 0.05), which was consistent with the superior bactericidal efficacy of SAEW in previous studies [22,23,24]. The microbial count decreased by 2.29 log_10_ CFU/g after treatment with 4 °C + SAEW, demonstrating a significantly stronger bactericidal effect compared to treatment at 25 °C + SAEW (decreased by 1.30 log_10_ CFU/g) (*p* < 0.05). Previous studies have hypothesized that low temperatures inhibit microbial activity and reduce the level of intercellular communication, affecting the development of biofilms [25], which might result in the easier penetration of SAEW and enhance the control of microorganisms. Overall, pretreatment with 4 °C + SAEW significantly reduced the microbial populations on oat seeds, providing an advantageous foundation for microbial control during subsequent germination treatments.

After soaking, the oat germination stage began. To further evaluate the effectiveness of SAEW spray in controlling microorganisms during the germination process, the sterilizing effect of temperature was investigated through comparisons with TW spray treatments (25 °C and 4 °C). As shown in Figure 1b, during the germination stage, the microbial populations on the surface of all treated samples showed an upward trend; however, all SAEW-pretreated groups maintained significantly lower microbial counts compared to the TW control group (*p* < 0.05). Additionally, the microbial populations of the 25 °C + SAEW pretreatment treatment group were significantly lower than those of the TW spray group after SAEW spraying (*p* < 0.05). A similar effect was observed in the 4 °C + SAEW pretreatment group. Previous studies showed that SAEW spraying significantly reduced microbial populations on soybean sprouts compared to TW treatments [26]. After 60 h of germination, the lowest microbial population count of 6.80 log_10_ CFU/g was recorded in the group treated with 4 °C + SAEW pretreatment + SAEW spray. This was 4.44 log_10_ CFU/g lower than the control group, 1.95 log_10_ CFU/g lower than 4 °C + SAEW pretreatment + TW spray, 1.59 log_10_ CFU/g lower than 25 °C + SAEW pretreatment + SAEW spray and 2.88 log_10_ CFU/g lower than 25 °C + SAEW pretreatment + TW spray. The results indicated that the 4 °C + SAEW pretreatment + SAEW spray treatment significantly reduced the microbial populations during oat germination compared to the control group and other treatments (*p* < 0.05), which could enhance the safety of oat sprouts and mitigate foodborne disease risks.

Oat sprout products were obtained when the oat had been germinated for 60 h, after which the oat sprout washing stage began. As shown in Figure 1c, the microbial population of the SAEW washing treatment group at different temperatures was significantly lower than that of the TW washing control group (11.30 log_10_ CFU/g) (*p* < 0.05), indicating that SAEW treatment had a favorable disinfection effect on oat sprouts, which is consistent with previous research results [27]. The microbial populations were detected at 5.75 log_10_ CFU/g in the 4 °C + SAEW washing treatment group after SAEW spray germination, representing reductions of 1.90, 1.71 and 2.96 log_10_ CFU/g compared to TW spray at 4 °C + SAEW washing, SAEW spray at 25 °C + SAEW washing and TW spray at 25 °C + SAEW washing, respectively. The results demonstrated that after washing the sprouts, the microbial control effect of 4 °C + SAEW treatment was significantly improved further (*p* < 0.05). Therefore, for the three stages of oat sprout production, 4 °C + SAEW treatment can achieve better microbial control effects.

**Figure 2:**
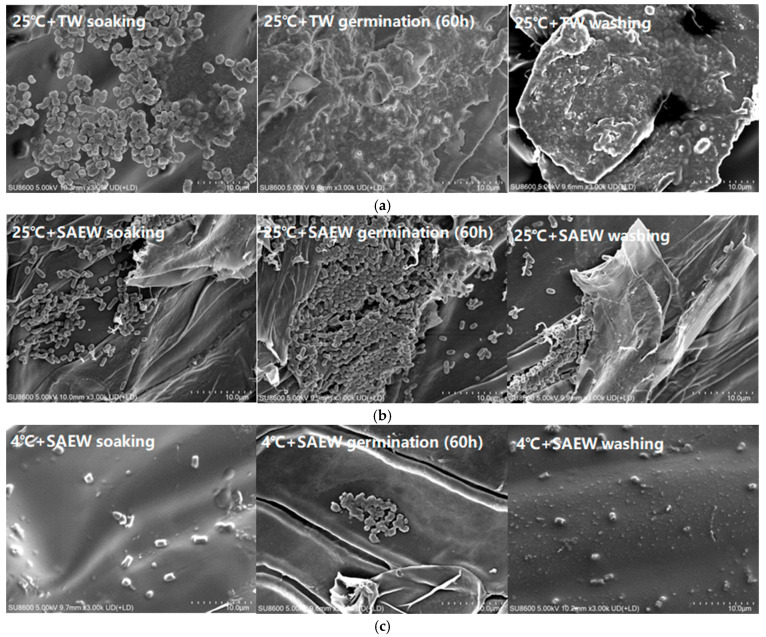
The scanning electron microscopy images of the surviving microbial population of oats inoculated by *E. coli* during the soaking, germination for 60 h and washing stages after being treated by 25 °C + TW (**a**), 25 °C + SAEW (**b**) and 4 °C + SAEW (**c**). The pH and available chlorine concentration (ACC) of the SAEW used in the experiment were 5.9 ± 0.1 and 30 mg/L, respectively; the tap water (TW) as the control was the local drinking water. The samples should be taken promptly after the completion of seed soaking, germination for 60 h and after the end of sprout washing.

The initial state (Figure S2) of the inoculated oat samples and the effects of different treatments on the microbial population were evaluated by SEM. As shown in Figure 2a, a large number of bacteria were still observed on the surface of oat samples soaked at 25 °C + TW control group, and the microbial populations were further substantially increased during the germination process. Compared to the control group, the microbial population counts on the surface of the SAEW-treated samples were significantly reduced throughout the entire oat sprout production process (*p* < 0.05), which was consistent with the aforementioned results. The microbial populations on the inoculated oat surfaces treated with the 4 °C + SAEW combination (Figure 2c) were also significantly lower than those treated with the 25 °C + SAEW combination (Figure 2b) (*p* < 0.05). Overall, the data obtained from SEM were further confirmed to support the conclusions described above.

### 3.2. Combined Effect of Low-Temperature Stress and SAEW for Reducing Natural Microflora During Oat Sprout Production

**Figure 3:**
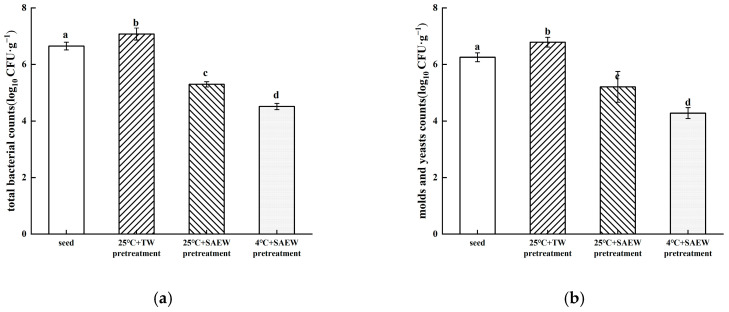
The total surviving bacterial counts (**a**) and mold and yeast counts (**b**) of oat seeds soaked with SAEW at different temperatures. The pH and available chlorine concentration (ACC) of SAEW used in the experiment were 5.9 ± 0.1 and 30 mg/L, respectively; the tap water (TW) as the control was the local drinking water. The samples should be taken promptly after the completion of seed soaking. Each value is expressed as the mean ± standard deviation of the three replicates. Different superscripts (a–d) show significant differences in the duration of germination (*p* < 0.05).

The effectiveness of low-temperature stress combined with SAEW to reduce natural microbial communities during the production and washing of oat sprouts was investigated. As shown in Figure 3a, before treatment, the total bacterial count of the initial microbial population in oat seeds was 6.65 log_10_ CFU/g. After soaking at 25 °C + TW, the total bacterial count increased to 7.08 log_10_ CFU/g. This increase was attributed to the moist environment provided by TW soaking, which promoted the growth and reproduction of natural bacterial colonies. Compared with the control group, the total bacterial count was significantly decreased after soaking in SAEW (*p* < 0.05), a finding consistent with previous studies on pea and buckwheat seeds soaked in SAEW [28,29]. Specifically, the total bacterial counts after soaking in 25 °C + SAEW and 4 °C + SAEW were decreased by 1.78 and 2.56 log_10_ CFU/g, respectively, compared to the control group. Similar results were observed for the microbial control of yeasts and molds in oat seeds by different treatments, as shown in Figure 3b. Compared with the yeast and mold population in the control, significant reductions occurred in both the 25 °C + SAEW and 4 °C + SAEW treatments (*p* < 0.05), with reductions of 1.57 and 2.50 log_10_ CFU/g, respectively. These results demonstrate that soaking in SAEW effectively reduces the total number of bacteria, molds and yeasts on the surface of oat seeds, with the 4 °C treatment showing superior efficacy.

**Figure 4:**
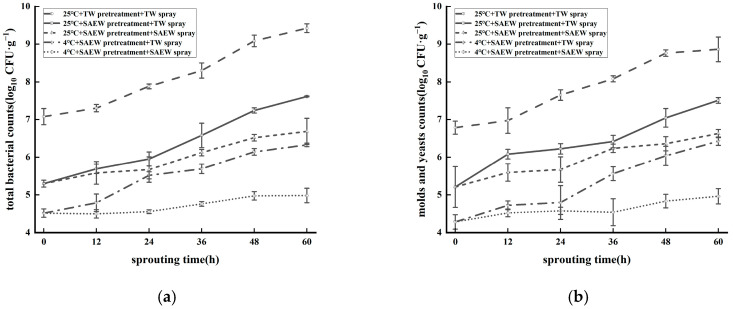
The total surviving bacterial counts (**a**) and mold and yeast counts (**b**) of oat seeds germinated with SAEW at different temperatures. The pH and available chlorine concentration (ACC) of SAEW used in the experiment were 5.9 ± 0.1 and 30 mg/L, respectively; the tap water (TW) as the control was the local drinking water. The samples should be taken promptly during germination (after 12, 24, 36, 48 and 60 h). Each value is expressed as the mean ± standard deviation of the three replicates.

The microbial control effect of different treatment groups on natural colonies at the stage of oat germination (Figure 4) was better than that of the 25 °C + TW pretreatment + TW spray control group. As shown in Figure 4a, with the extension of the germination time, the total bacterial counts in both the control and treatment groups increased gradually; however, the increase in the total bacterial counts in the two SAEW spray treatment groups was significantly lower than that in the TW spray treatment group (*p* < 0.05). Previous studies observed that the total bacterial count of sprouts cultured with SAEW was significantly lower than that of sprouts cultured with TW after a period of germination [30,31]. After 60 h of germination, the 4 °C + SAEW pretreatment + SAEW spray treatment group exhibited the lowest microbial population count at 4.98 log_10_ CFU/g. This value was 4.44 log_10_ CFU/g lower than that of the control group, 1.35 log_10_ CFU/g lower than the 4 °C + SAEW pretreatment + TW spray group, 1.70 log_10_ CFU/g lower than the 25 °C + SAEW pretreatment + SAEW spray group and 2.63 log_10_ CFU/g lower than the 25 °C + SAEW pretreatment + TW spray group. As shown in Figure 4b, during the germination stage, the total number of molds and yeasts in oat sprouts followed a similar trend. After 60 h of germination, the lowest microbial population count (4.96 log_10_ CFU/g) was recorded in the 4 °C + SAEW pretreatment + SAEW spray treatment group, compared to the microbial counts in the control group, 4 °C + SAEW pretreatment + TW spray group, 25 °C + SAEW pretreatment + SAEW spray group and 25 °C + SAEW pretreatment + TW spray group, which were reduced by 3.90, 1.27, 1.67 and 2.55 log_10_ CFU/g, respectively. The results demonstrated that the control effect of the 4 °C + SAEW spray treatment on the natural microbial population during germination was significant (*p* < 0.05), and it will not affect the morphology of oat seeds (Figure S1), establishing a foundation for the preparation of oat sprout products.

**Figure 5:**
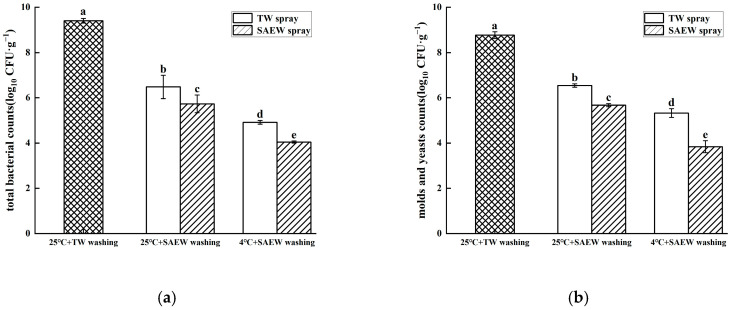
The total surviving bacterial counts (**a**) and mold and yeast counts (**b**) of oat seeds cleaned with SAEW at different temperatures. The pH and available chlorine concentration (ACC) of SAEW used in the experiment were 5.9 ± 0.1 and 30 mg/L, respectively; the tap water (TW) as the control was the local drinking water. The samples should be taken promptly after sprout washing ends. Each value is expressed as the mean ± standard deviation of the three replicates. Different superscripts (a–e) show significant differences in the duration of germination (*p* < 0.05).

At the end of the 60 h germination period, oat sprouts from the different treatments were obtained, washed and analyzed for natural microbial populations of bacteria, mold and yeast. As shown in Figure 5a, compared with the 25 °C + TW washing control group, the total bacterial count in all SAEW washing treatment groups showed significantly reductions (*p* < 0.05), consistent with previous findings in buckwheat sprouts [28]. After germination with SAEW spray, the total bacterial count detected in the 4 °C + SAEW washing treatment group was 4.05 log_10_ CFU/g, representing a 0.88 log_10_ CFU/g reduction compared to TW spray at 25 °C + SAEW. These results demonstrate that under identical washing conditions, the total bacterial counts in oat sprouts treated with SAEW spray were significantly lower than that of TW spray treatment (*p* < 0.05). Similarly, the total bacterial count in the 4 °C + SAEW washing treatment group was significantly lower than that in other treatment groups (*p* < 0.05), with reductions of 1.69 and 2.44 log_10_ CFU/g compared to SAEW spray at 25 °C + SAEW and TW spray at 25 °C + SAEW washing, respectively. These findings confirm that SAEW treatment significantly reduced bacterial populations on sprouts across temperature conditions, with the 4 °C + SAEW spray + SAEW washing combination being most effective. As shown in Figure 5b, the overall microbial control effect of different treatments on the total number of molds and yeasts in harvested oat sprouts was similar to the former. The microbial populations were detected at 3.84 log_10_ CFU/g in the 4 °C + SAEW washing treatment group after SAEW spray germination, compared to the TW spray at 4 °C + SAEW, SAEW spray at 25 °C + SAEW and TW spray at 25 °C + SAEW washing treatment groups, which were reduced by 1.99, 1.83 and 2.71 log_10_ CFU/g, respectively. Therefore, for the three stages of oat sprout production, 4 °C + SAEW treatment provided better microbial control effects on natural microbial populations.

Although the above results indicate that the 4 °C + SAEW treatment has favorable microbial control effects in the production process of oat sprouts, this study still has certain limitations and needs further research. For instance, it only preliminarily verified the bactericidal effect of the combined treatment of 4 °C and SAEW, but did not systematically explore the interaction between different temperature gradients and the effective concentration of SAEW. In the future, further optimization of parameter combinations is needed to establish a temperature concentration and time dynamic model. A low-temperature environment may promote the survival of some cold-tolerant microorganisms, and the resistance mechanism of cold-tolerant bacteria needs to be analyzed through metagenomics in the future. SAEW needs to be produced and used in the field. Its stability is limited by ambient temperature and storage conditions, and it remains to be explored how to achieve the continuous preparation and precise dosing of SAEW in industrial production.

## 4. Conclusions

In the present study, the control effects of 25 °C + TW control, 25 °C + SAEW and 4 °C + SAEW treatments on microorganisms during the entire process of oat malt production (including soaking, germination and washing) were compared using the plate counting method. The results showed that in all treatment groups, the 4 °C + SAEW treatments had the lowest number of residual colonies. Compared with the control group, the detection rates of *Escherichia coli* in the three stages after 4 °C + SAEW treatment decreased by 2.32, 4.44 and 5.55 log_10_ CFU/g, respectively. The detection rates of the total bacterial count decreased by 2.56, 4.43 and 5.36 log_10_ CFU/g, respectively. The detection rates of molds and yeasts decreased by 2.50, 3.90 and 5.22 log_10_ CFU/g, respectively. This indicates that the microorganisms were effectively controlled by the 4 °C + SAEW treatment. This result was demonstrated by SEM. However, the effectiveness in industrial conditions still needs to be further verified. In conclusion, the 4 °C + SAEW treatment has great potential in commercial sprout production and food safety improvement. 

## Data Availability

The original contributions presented in this study are included in the article/Supplementary Materials; further inquiries can be directed to the corresponding authors.

## Supplementary Materials

The following supporting information can be downloaded at: https://www.mdpi.com/article/10.3390/foods14071083/s1, Figure S1: The changes in the morphology of oats treated with slightly acidic electrolytic water (SAEW) during germination. The pH and available chlorine concentration (ACC) of SAEW used in the experiment were 5.9 ± 0.1 and 30 mg/L, respectively; the tap water (TW) as the control was the local drinking water. The samples were taken at 0, 12, 24, 36, 48 and 60 h of germination. Figure S2. The scanning electron microscopy images of untreated surviving microbial populations in oats inoculated with *E. coli*.
