# Supplementary material for: Combined Effect of Low-Temperature Stress and Slightly Acidic Electrolyzed Water (SAEW) on the Microbial Control of Oat Sprout Production
Source: Foods. 2025 Mar 21;14(7):1083. doi: 10.3390/foods14071083 (PMC11988572; doi:10.3390/foods14071083)
Supplement: Supplementary file 1 [file foods-14-01083-s001.zip › foods-3510737-supplementary.pdf]

Supplementary Material

Combined effect of Low temperature stress and slightly acidic electrolyzed water (SAEW) on the microbial control of oat sprouts production

Shaokang Liu, Hongrui Ren, Lin Chen, Tongjiao Wu*, Jianxiong Hao*

College of Food Science and Biology, Hebei University of Science and Technology, Shijiazhuang 050018, China

***Corresponding authors.**

Tel./fax: +03 11 81668463.

E-mail addresses: cauhjx@163.com (J. Hao); katherine_0827@163.com (T. Wu).

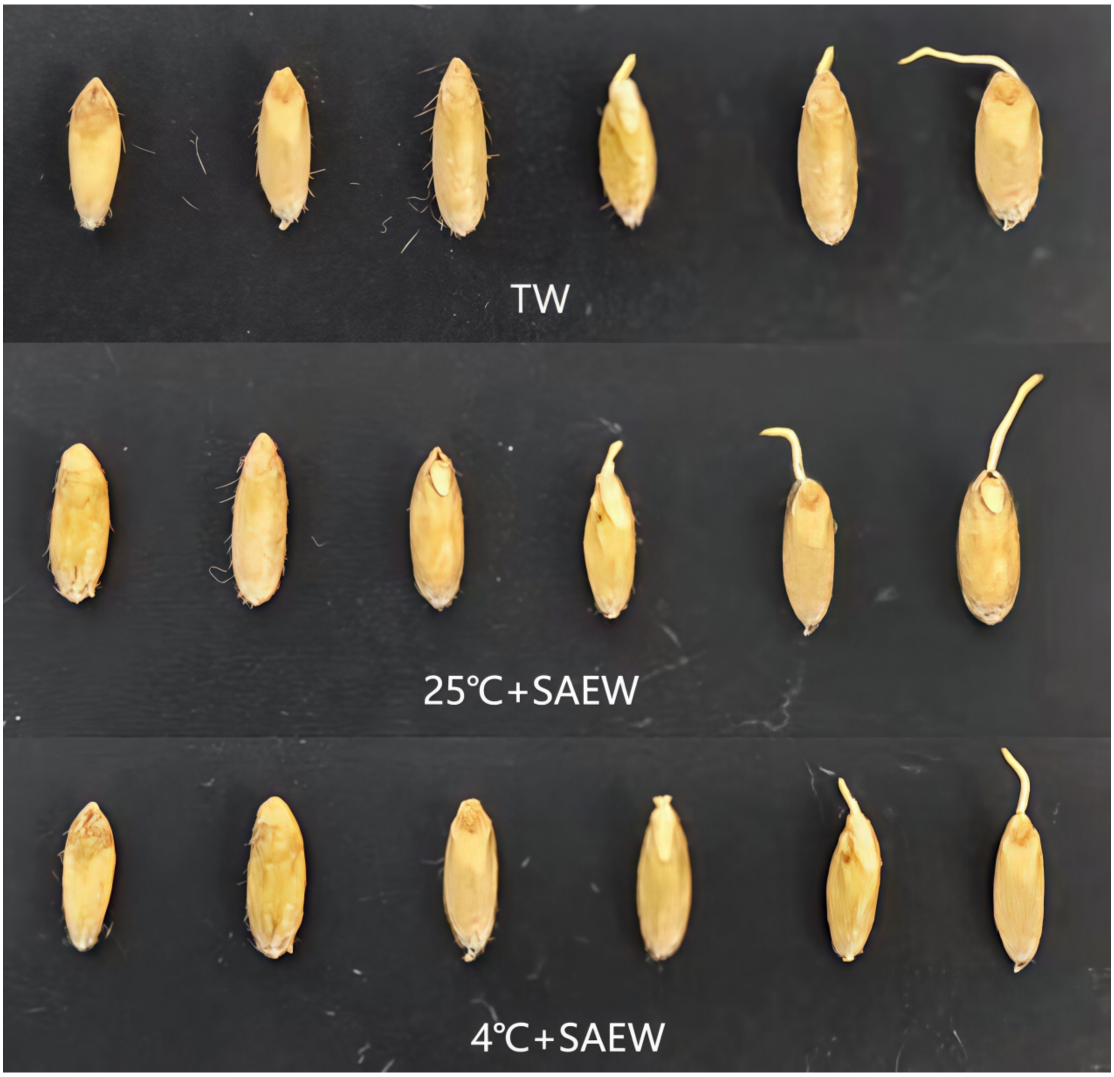

Figure S1. The morphological changes of oats during germination were treated with slightly acidic electrolyzed water (SAEW) at different temperatures. The pH and available chlorine concentration (ACC) of SAEW used in the experiment were 5.9 ± 0.1 and 30 mg/L, respectively; the tap water (TW) as control was the local drinking water. Samples were taken at 0, 12, 24, 36, 48 and 60 h of germination.

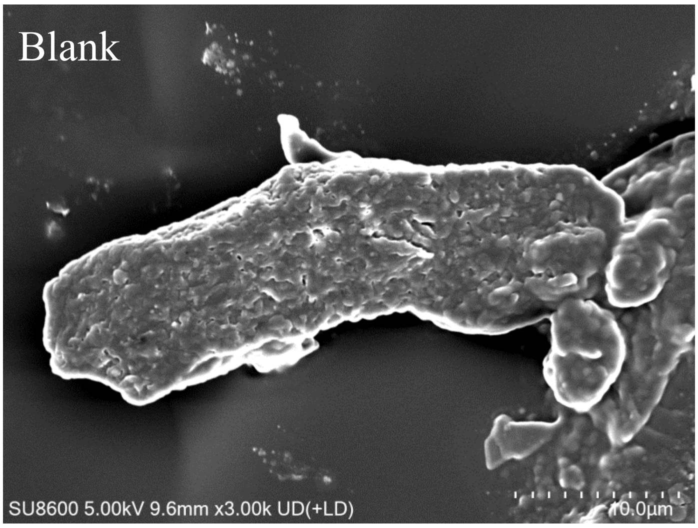

Figure S2. The scanning electron microscopy images of untreated surviving microbial populations in oats inoculated with *E. coli*.
